# Lupus podocytopathy and antiphospholipid syndrome in a child with SLE: A case report and literature review

**DOI:** 10.3389/fped.2022.950576

**Published:** 2022-08-19

**Authors:** Guo-min Li, Yi-fan Li, Qiao-qian Zeng, Xiao-mei Zhang, Hai-mei Liu, Jia-yan Feng, Yu Shi, Bing-bing Wu, Hong Xu, Li Sun

**Affiliations:** ^1^National Children's Medical Center, Shanghai, China; ^2^Department of Rheumatology, Children's Hospital of Fudan University, Shanghai, China; ^3^Department of Pathology, Children's Hospital of Fudan University, Shanghai, China; ^4^Medical Transformation Centre, Children's Hospital of Fudan University, Shanghai, China; ^5^Department of Nephrology, Children's Hospital of Fudan University, Shanghai, China

**Keywords:** antiphospholipid syndrome, foot process effacement, lupus podocytopathy, systemic lupus erythematosus, aPL antibodies

## Abstract

Lupus podocytopathy is a glomerular lesion in systemic lupus erythematosus (SLE) characterized by diffuse podocyte foot process effacement (FPE) without immune complex (IC) deposition or with only mesangial IC deposition. It is rarely seen in children with SLE. A 13-year-old girl met the 2019 European League Against Rheumatism (EULAR)/ American College of Rheumatology (ACR) Classification Criteria for SLE based on positive ANA; facial rash; thrombocytopenia; proteinuria; and positive antiphospholipid (aPL) antibodies, including lupus anticoagulant (LAC), anti-β2 glycoprotein-I antibody (anti-β2GPI), and anti-cardiolipin antibody (aCL). The renal lesion was characterized by 3+ proteinuria, a 4.2 mg/mg spot (random) urine protein to creatinine ratio, and hypoalbuminemia (26.2 g/l) at the beginning of the disease. Kidney biopsy findings displayed negative immunofluorescence (IF) for immunoglobulin A (IgA), IgM, fibrinogen (Fb), C3, and C1q, except faint IgG; a normal glomerular appearance under a light microscope; and diffuse podocyte foot process effacement (FPE) in the absence of subepithelial or subendothelial deposition by electron microscopy (EM). Histopathology of the epidermis and dermis of the pinna revealed a hyaline thrombus in small vessels. The patient met the APS classification criteria based on microvascular thrombogenesis and persistently positive aPL antibodies. She responded to a combination of glucocorticoids and immunosuppressive agents. Our study reinforces the need to consider the potential cooccurrence of LP and APS. Clinicians should be aware of the potential presence of APS in patients with a diagnosis of LP presenting with NS and positivity for aPL antibodies, especially triple aPL antibodies (LCA, anti-β2GPI, and aCL).

## Introduction

The podocyte is a visceral epithelial cell that is the core component of the glomerular filtration barrier (1). Podocytopathies are kidney diseases in which direct or indirect podocyte injury drives proteinuria or nephrotic syndrome (2, 3). The most frequent histopathologic findings of primary injury to podocytes are minimal change disease (MCD) and focal segmental glomerulosclerosis (FSGS) (2–4). However, MCD and FSGS are morphologic descriptions that can be caused by various pathogenic pathways (2, 4). Systemic lupus erythematosus (SLE) is a chronic immune complex-mediated disease characterized by disseminated inflammation that may affect multiple organs (5). Renal nephritis (LN) occurs in 30 to 70% of patients with SLE, and there is compelling evidence to suggest that glomerular epithelial cells, and podocytes in particular, are also involved in glomerular injury in patients with SLE (6–8). Lupus podocytopathy (LP) is also a glomerular lesion in SLE characterized by proteinuria or nephrotic syndrome (NS), demonstrating MCD, mesangial proliferation (MsP) or FSGS. Meanwhile, by electron microscopy (EM), diffuse podocyte FPE is observed in the absence of subepithelial or subendothelial deposition (9–11). Antiphospholipid syndrome (APS) is an autoimmune disease characterized by the occurrence of venous and/or arterial thrombosis and pregnancy morbidity in the presence of pathogenic autoantibodies known as antiphospholipid (aPL) antibodies (12, 13). APS may be associated with other diseases, mainly SLE. Herein, we report a child suffering from SLE with LP and APS.

## Case presentation

**Figure 1:**
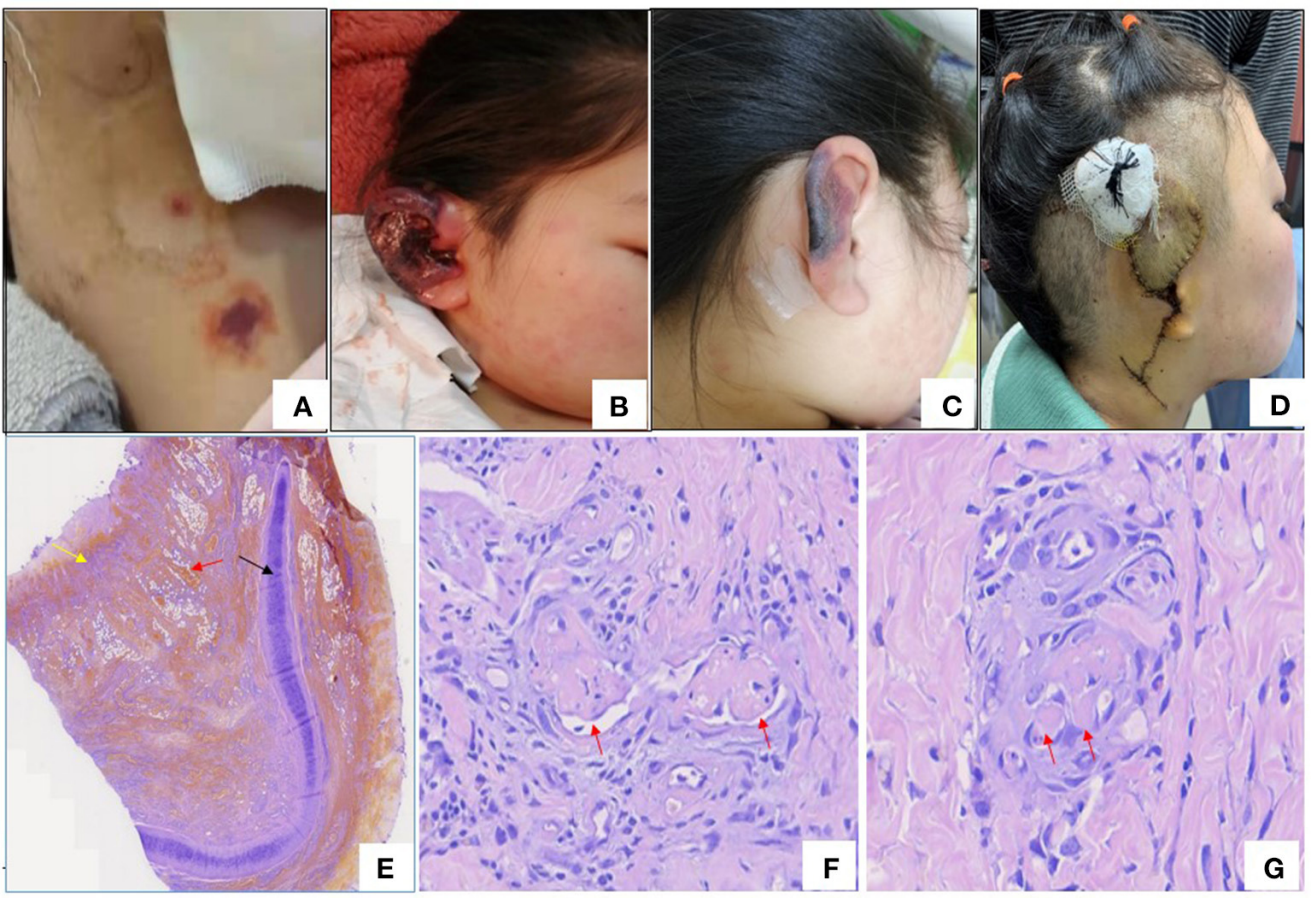
**(A)** Rash in neck. **(B)** Congestion and edema of right auricle. **(C)** Necrosis of right auricle. **(D)** Right auricle excision and flap transplantation. **(E)** Histopathology of right ear (Hematoxylin –Eosin staining×40), black arrow: cartilage, yellow arrow: necrosis and red arrow: necrosis and red arrow: hemorrhage. **(F,G)** The histopathologic examination of skin of right ear revealed microthrombi in small vessel (red arrows) (Hematoxylin-Eosin staining).

The patient, a 13-year-old Chinese girl, presented with fever and persistent right upper abdominal pain in Sep 2020. She was admitted to the surgery department of her local hospital, and computed tomography (CT) of the upper abdomen revealed cholecystitis. Laboratory findings suggested thrombocytopenia (77.0 × 10^9^/l, normal range 100–300 × 10^9^/l), mildly elevated serum (91 U/l, normal range 40–85 U/l) and urine (1,329 U/l, normal range 400–1,000 U/l) amylase, abnormal alanine aminotransferase (ALT; 72 U/l, normal range 7–40 U/l) and aspartate aminotransferase (AST; 42 U/l, normal range 13–35 U/l), elevated D-dimer (4,140 ng/ml, normal range 0–500 ng/ml), and positive anti-nuclear antibodies (ANA; 1:320) and perinuclear antineutrophil cytoplasmic antibody (p-ANCA; 1:10). She was diagnosed with cholecystitis and acute biliary pancreatitis and was treated with antibiotics and supportive treatment, such as fasting, rehydration, parenteral nutrition, and anticoagulation. Although the symptoms were relieved after treatment, the thrombocytopenia did not improve. However, the persistent thrombocytopenia and positive ANA in the patient did not attract sufficient attention from surgeons, and the symptoms of fever and abdominal pain relapsed after 2 months. She was readmitted to the surgery department of her local hospital. Magnetic resonance cholangiopancreatography (MRCP) was normal, abdominal ultrasound revealed that the gallbladder wall was rough and thickened, and lung CT showed pleural and pericardial effusion. Laboratory findings revealed thrombocytopenia (52.0 × 10^9^/l, normal range 100–300 × 10^9^/l), proteinuria (3+), decreased serum albumin (26.2 g/l, normal range 40–55 g/l), low levels of complement 4, and positive ANA (1:320). The spot (random) urine protein to creatinine ratio (UPr/Cr ratio) was 4.2 mg/mg (normal <0.2 mg/mg), which suggests nephrotic range proteinuria (UPr/Cr ratio>2.0 mg/mg). She was diagnosed with cholecystitis and was treated with antibiotics. The abdominal pain was relieved, but the fever was not improved. Moreover, she had a rash (Figures 1A,B) in face and neck accompanied by swelling and pain of the right auricle (Figure 1B), which became necrotic (Figure 1C). The right auricle had to be removed, and new skin had to be grafted on from her neck (Figure 1D). Pathological changes from the auricle and its surrounding skin displayed microvascular thrombogenesis (Figures 1E–G). She was treated with oral prednisolone (2 mg/kg/day) and aspirin because she may have had connective tissue disease and a hypercoagulable state after a physician consultation. The fever was relieved after treatment with prednisolone, and the proteinuria was reduced.

**Figure 2:**
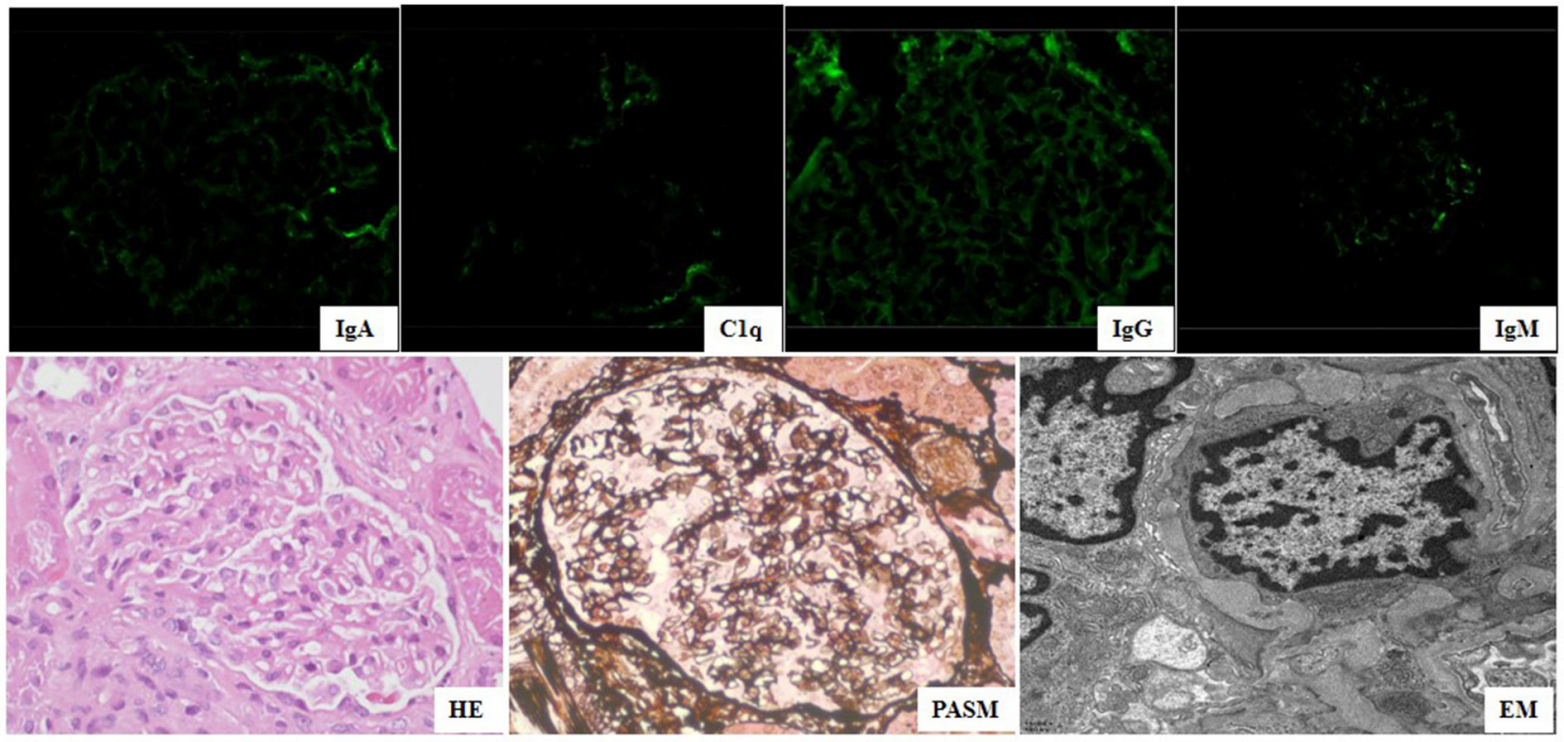
Kidney biopsy of findings showed negative IgA, IgM, Clq, and faint IgG under immunofluorescence (×400) microscopy, normal glomerular change (HE&PASM: periodic acid-silver metheramine) under light microscopy (×400), diffuse podocyte foot process effacement (FPE) in absence of sub-epithelial or sub-endothelial deposition under electron microscopy (×11,600).

She was referred to our hospital for evaluation because of persistent thrombocytopenia and proteinuria. Physical examination revealed a rash on the face and neck, absence of the right pinna, and surgical scarring on the area of the right pinna. Other physical findings were unremarkable. Her medical history and family history were unremarkable. Laboratory testing showed mild anemia (hemoglobin 95 g/l, normal range 110–160 g/l), 2 + proteinuria (0.96 g/24 h, normal <0.15 g/ 24 h), mildly elevated ALT and AST, and decreased serum albumin (34.2 g/L, normal range 39–45 g/L). The UPr/Cr ratio was 2.2 mg/mg. Autoantibodies were positive for ANA (1:640), p-ANCA (1:10), lupus anticoagulant (LAC), anti-β2 glycoprotein-I antibody (anti-β2GPI), anti-cardiolipin antibody (aCL) immunoglobulin G (IgG) and immunoglobulin M (IgM), and the others were negative. Kidney biopsy was performed because of persistent haematuria and proteinuria, and the findings displayed negative immunofluorescence (IF) for immunoglobulin A (IgA), IgM, fibrinogen (Fb), C3, and C1q, except faint IgG (Figure 2); a normal glomerular appearance under light microscopy (Figure 2); and diffuse podocyte foot process effacement in the absence of subepithelial or subendothelial deposition on EM (Figure 2). Whole exon sequencing (WES) in the core family was performed, and no disease-causing mutations in any genes associated with SLE or APS were detected. The patient was diagnosed with SLE accompanied by LP and APS.

She was treated with intravenous pulse methylprednisolone followed by oral prednisolone (60 mg/day) and hydroxychloroquine (5 mg/kg/day) combined with monthly intravenous cyclophosphamide (CTX) pulses (500 mg/m^2^) as induction therapy. Other treatments included oral enalapril and subcutaneous injection of low molecular weight heparin sodium for 2 weeks followed by oral aspirin. After 6 pulses of CTX, she started maintenance therapy with mycophenolate mofetil (MMF) and continued to gradually taper doses of oral prednisolone. The proteinuria gradually decreased, and she achieved clinical remission and complete remission at 2 and 7 months of follow-up, respectively. Although the aCL antibody turned negative soon after treatment, LAC and anti-β2GPI were persistently positive (Table 1).

**Table 1:** Coagulation function and aPL antibodies in the patient during following up.

**Testing items**	**29/1/2021**	**17/2/2021**	**9/3/2021**	**12/5/2021**	**9/7/2021**	**11/9/2021**	**15/11/2021**	**Normal ranges**
D-dimer	4.4	1.98	0.63	0.45	0.76	0.89	0.33	0–0.5
FIB(g/L)	7.44	3.90	1.95	4.04	3.54	2.93	2.87	2–4
FDP(g/L)	20.01	8.51	1.28	1.54	1.19	1.30	0.10	0–5
RLACST	3.57	2.68	1.58	1.31	1.54	1.15	1.53	0.8–1.2
Anti-β2GP1(RU/ml)*	82.2	45.0	50.7	47.8	26.9	39.8	26.9	<20
aCL-IgA*	4	<2	<2	<2	<2	5.5	<2	<12-PL-IgA
aCL-IgG*	14.6	2.5	2.6	2.2	<2	2.4	2.5	<12-PL-IgG
aCL-IgM*	16.3	5.9	10.7	4.5	4.7	3.9	4.0	<12-PL-IgM

* Was tested by Enzyme-Linked ImmunoSorbent Assay (ELISA).

FIB, Fibrinogen; FDP, fibrin degradation product; RLACST, Ratio of lupus anticoagulant screening test; anti-β2GP1, anti-β2 glycoprotein-1 antibody; aCL, anti-cardiolipin antibody.

Informed written consent was obtained from the patient's parents for publication of this case report and accompanying images. Ethics board approval and consent were obtained for this work from the Ethics Committee at the Children's Hospital of Fudan University, Shanghai, China.

## Discussion and conclusion

Herein, we report an SLE patient complicated with LP and APS. The patient met the 2019 European League Against Rheumatism/American College of Rheumatology Classification Criteria for SLE (14) based on positive ANA (1:640); facial rash (six points); thrombocytopenia (four points); proteinuria (4 points); and positive aPL antibodies (two points), including LAC, anti-β2GP1, aCL antibody IgG and IgM. The renal lesion presented with NS: 3+ proteinuria, a 4.2 mg/mg UPr/Cr ratio, and hypoalbuminemia (26.2 g/l) at the beginning of the disease. The patient presented with acute abdominal pain as the first symptom, which led to a misdiagnosis of acute abdominal disease in the surgery department of the local hospital. Abdominal pain should be vigilant against gastrointestinal vasculitis in children with SLE.

LN has a large impact on SLE prognosis, resulting in a risk of end-stage kidney disease (ESKD) of 10% after 5 years of follow-up (15, 16). It is thought to be a typical immune-complex-mediated kidney disease that displays a “full house” model on IF and various electron dense deposits in the mesangium, subepithelial or subendothelial regions (6, 17). However, LP is characterized by diffuse epithelial cell FPE without immune complex deposition or with only mesangial immune complex deposition (9–11). It is a newly emerging entity of non-immune complex-mediated lupus nephropathy and is not yet included in the updated 2018 International Society of Nephrology/Renal Pathology Society (ISN/RPS) classification of LN (18, 19). LP was coined by Kraft in 2005 when he described eight SLE patients with nephrotic range proteinuria and diffuse FPE on EM but no immune deposits in glomerular capillaries or endocapillary proliferation of glomeruli (20). Although there are no formalized guidelines for the diagnosis of LP, the diagnostic criteria established by Hu were taken as the most commonly used diagnostic criteria of LP, which are the following: clinical presentation of NS or nephrotic range proteinuria in a patient with SLE; kidney biopsy findings of normal glomeruli or minimal change disease (MCD) or focal segmental glomerulosclerosis (FSGS) (with or without mesangial proliferation) on light microscopy and diffuse and severe FPE on EM; and the absence of subendothelial or subepithelial immune deposits on light, IF, and electron microscopy (9). The renal lesions in our patient met the diagnostic criteria for LP established by Hu based on nephrotic range proteinuria; MCD without mesangial proliferation on light microscopy and diffuse FPE on EM; and the absence of immune deposits under the endothelium and epithelium by light, IF, and electron microscopy.

LP accounts for 1.33% of all LN biopsies in adults (9), representing 8.14% in children (21). Our data showed LP in <1% of all children with LN biopsies (unpublished). To date, eleven pediatric cases of LP have been reported in PubMed. Four were case reports including our patient (Table 2), and a cohort study included seven patients (21–24), which suggests that it is a very rare complication of SLE in children. All children with LP have been girls and present with NS (21–24). Kidney biopsy findings revealed MCD in eight patients and FSGS in three patients (21–24). All patients achieved remission after treatment with a combination of prednisolone and immunosuppressive agents, including mycophenolate mofetil (MMF, n = 4), CTX (*n* = 3), cyclosporine (*n* = 2), and azathioprine (*n* = 2) (21–24). One of these 11 patients (patient 2) with FSGS resisted prednisolone treatment alone, but she responded to a combination of prednisolone and cyclosporine, which was switched to MMF because of posterior reversible encephalopathy syndrome (PRES) associated with cyclosporine treatment (23). As of the time of case reporting, three patients relapsed (21). Thirty-four percent of patients with LP developed acute kidney injury (AKI) in adults, but no AKI was reported in children with LP (9, 21–24).

**Table 2:** clinical features of SLE cases with LP in children.

**Case**	**Gender**	**Age at diagnosis (years)**	**Renal manifest ations**	**Extra-renal manifestations**	**Positive auto-antibody**	**Pathologic changes**	**Immunosuppressive therapy**		**Treatment response**
							**Induction**	**Maintenance**	
1. Wang et al. (22)	Female	14.1	Nephrotic syndrome	Fever, abdominal pain, arthritis, cutaneous lesions	ANA Anti-dsDNA	MCD	Prednisolone CTX	Prednisolone	Remission
2. Ito et al. (23)	Female	11	Nephrotic syndrome	Fever, cutaneous lesions, Raynaud's phenomenon, hematologic involvement	ANA Anti-dsDNA	FSGS	Prednisolone Cyclosporine/MMF	Prednisolone MMF	Remission
3. Pilania et al. (24)	Female	3	Nephrotic syndrome	Alopecia, chylous ascites, serositis, Hematologic and neurological involvement	ANA Anti-dsDNA	MCD	Prednisolone CTX	Prednisolone MMF	Remission
4	Female	13.2	Nephrotic syndrome	Fever, abdominal pain, serositis, cutaneous lesions, hematologic involvement, APS	ANA, anti-β2GPI, aCL, LCA.	MCD	Prednisolone CTX	Prednisolone MMF	Remission
Groups (seven cases) Abdelnabi (21)	Female	13.60 ± 2.30	Nephrotic syndrome	-	-	MCD(*N* = 5)/FSGS(*N* = 2)	Prednisolone MMF (*N* = 3) Aza (*N* = 2) CsA (*N* = 2)	-	Remission

ANA, anti-nuclear antibodies; Anti-dsDNA, anti-double stranded DNA; APS, antiphospholipid syndrome; Aza, azathioprine; CsA, cyclosporine A; CTX, cyclophosphamide; FSGS, focal segmental glomerulosclerosis; MMF, mycophenolate mofetil; N, numbers; RP, Raynaud's phenomenon.

APS is an autoimmune disease characterized by the occurrence of venous and/or arterial thrombosis and pregnancy morbidity in the presence of pathogenic autoantibodies known as aPL, including aCL, anti-β2GPI antibodies and LCA (12, 25). APS may be associated with other diseases, mainly SLE. The prevalence of aCL, anti-β2GPI and LCA antibodies was 44, 40, and 22% in children with SLE, respectively (26). It has been estimated that APS may develop in up to 50–70% of patients with both SLE and aPL after 20 years of follow-up (27). Our patient met the current consensus criteria for APS based on microvascular thrombosis in the auricle and positive anti-β2GPI antibodies and LCA lasting for more than 12 weeks. Both APS and SLE have renal involvement. The former is characterized by hypertension, renal artery stenosis, renal infarction, and renal vein thrombosis, and the latter can reveal proteinuria, haematuria and hypertension and sometimes renal failure (27–29). Thus, renal involvement in the patient is caused by SLE. To the best of our knowledge, the coexistence of APS and LP in the same patient with SLE has not been reported in children. However, comorbidities of APS and LP in adults with SLE are very rare (30, 31).

In conclusions, LP is rarely described in children with SLE, and it responds to a combination of glucocorticoids and immunosuppressive agents. Our study reinforces the need to consider the potential co-occurrence of APS and LP. Clinicians should be aware of the potential presence of APS in patients with a diagnosis of LP presenting with NS and positivity for aPL antibodies, especially triple aPL antibodies (LCA, aCL, and anti-β2GPI).

## Data availability statement

The original contributions presented in the study are included in the article/supplementary material, further inquiries can be directed to the corresponding author/s.

## Ethics statement

The studies involving human participants were reviewed and approved by the Ethics Committee at the Children's Hospital of Fudan University, Shanghai, China. Written informed consent to participate in this study was provided by the participants' legal guardian/next of kin.

## Author contributions

G-mL, Y-fL, Q-qZ, J-yF, YS, and B-bW performed the experiments and collected data. X-mZ, H-mL, and HX analyzed the data. G-mL wrote the manuscript. LS conceived and supervised the project. All authors contributed to the article and approved the submitted version.

## Conflict of interest

The authors declare that the research was conducted in the absence of any commercial or financial relationships that could be construed as a potential conflict of interest.

## Publisher's note

All claims expressed in this article are solely those of the authors and do not necessarily represent those of their affiliated organizations, or those of the publisher, the editors and the reviewers. Any product that may be evaluated in this article, or claim that may be made by its manufacturer, is not guaranteed or endorsed by the publisher.
